# Supplementary material for: Inhibiting the copper efflux system in microbes as a novel approach for developing antibiotics
Source: PLoS One. 2019 Dec 30;14(12):e0227070. doi: 10.1371/journal.pone.0227070 (PMC6936879; doi:10.1371/journal.pone.0227070)
Supplement: S1 File — (PDF) [file pone.0227070.s001.pdf]

Model structures of apo and holo conformations of CusB based on the DEER constrains

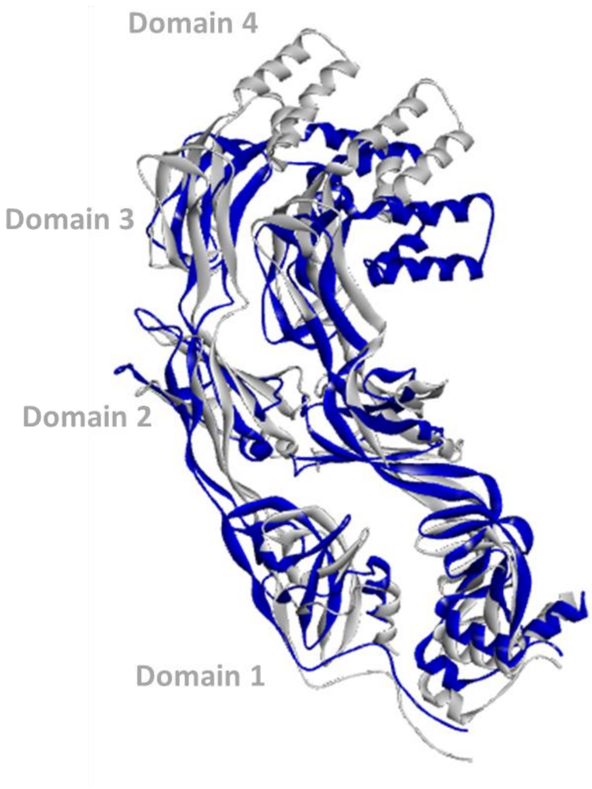

Fig A: Model structures of apo and holo conformation of CusB. A ribbon view of the apo CusB model structure (gray) overlaid on the holo model structure (blue) based on DEER constrains (Meir et al. 2017).

Validation of the peptides stability using ESI-MS

In order to confirm the stability of the peptides, them were diluted to 150 mM solution in double-distilled water, then incubated at 37 ºC for 24 hr. ESI-MS spectra for the various peptides are presented in Figure S2 before the incubation (Figure S2A) and after the incubation (Figure S2B). All peptides besides pep1 were found to be stable at the given conditions.

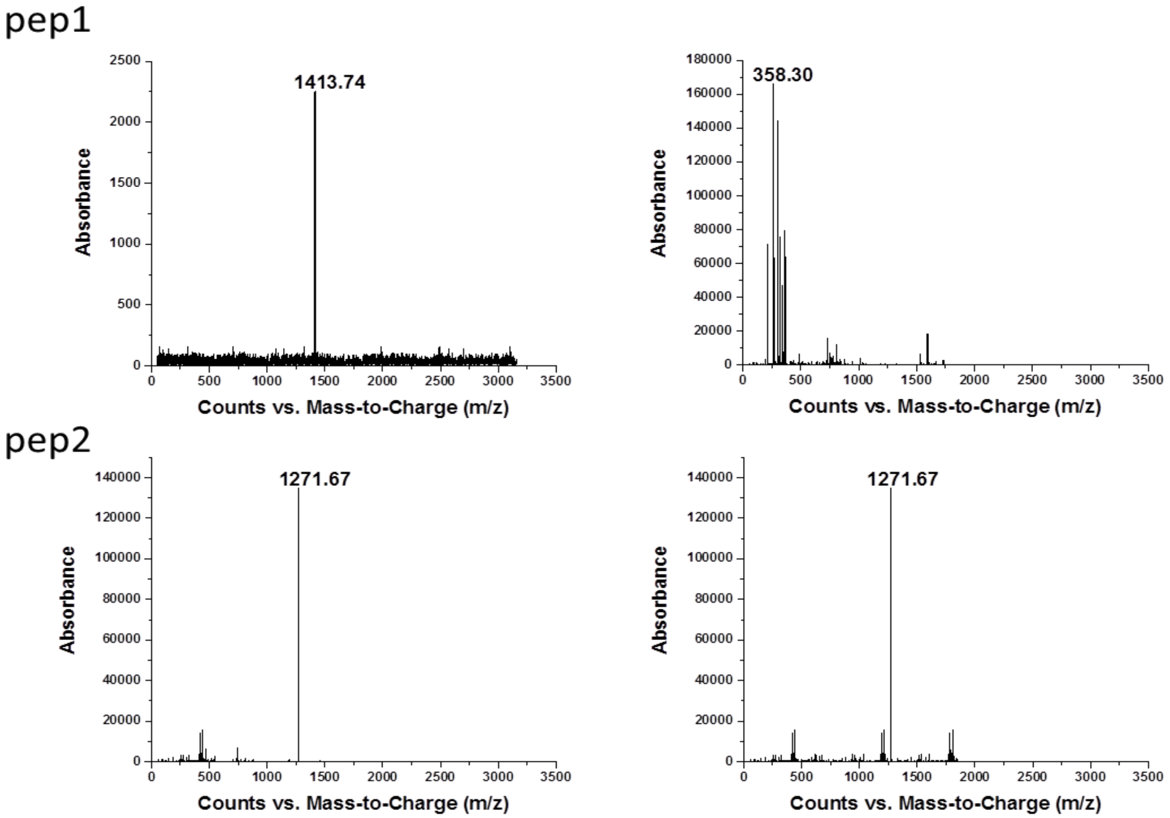

Fig B: ESI-MS. ESI-MS spectra for pep1 and pep2, left panel presents the spectra after peptide synthesis, right panel shows spectra after incubation at 37 ºC for 24 hr.

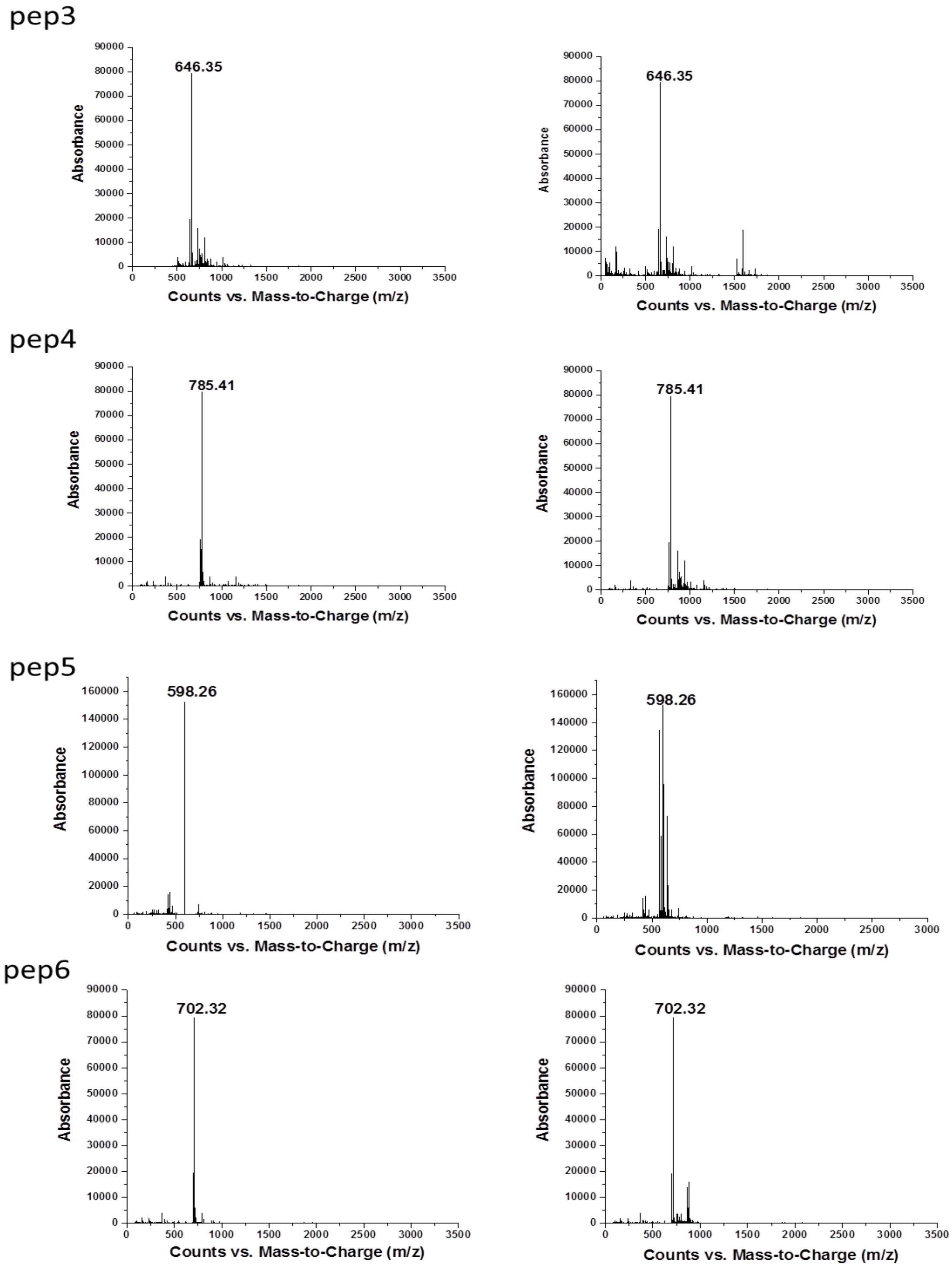

Fig C: ESI-MS. ESI-MS spectra for pep3, pep4, pep5 and pep6, left panel presents the spectra after peptide synthesis, right panel shows spectra after incubation at 37 ºC for 24 hr.

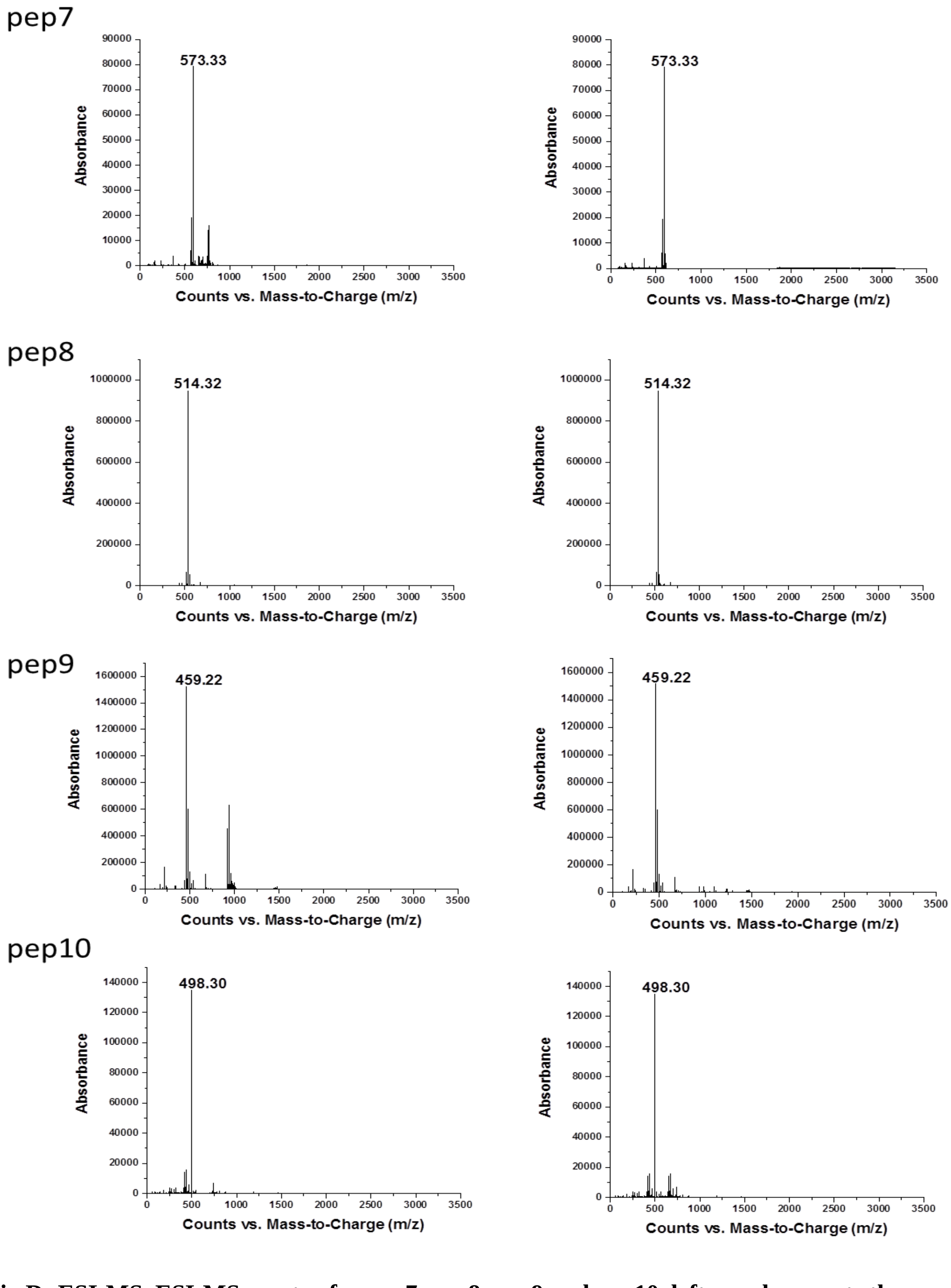

Fig D: ESI-MS. ESI-MS spectra for pep7, pep8, pep9 and pep10, left panel presents the spectra after peptide synthesis, right panel shows spectra after incubation at 37 ºC for 24 hr.

Growth rates experiments

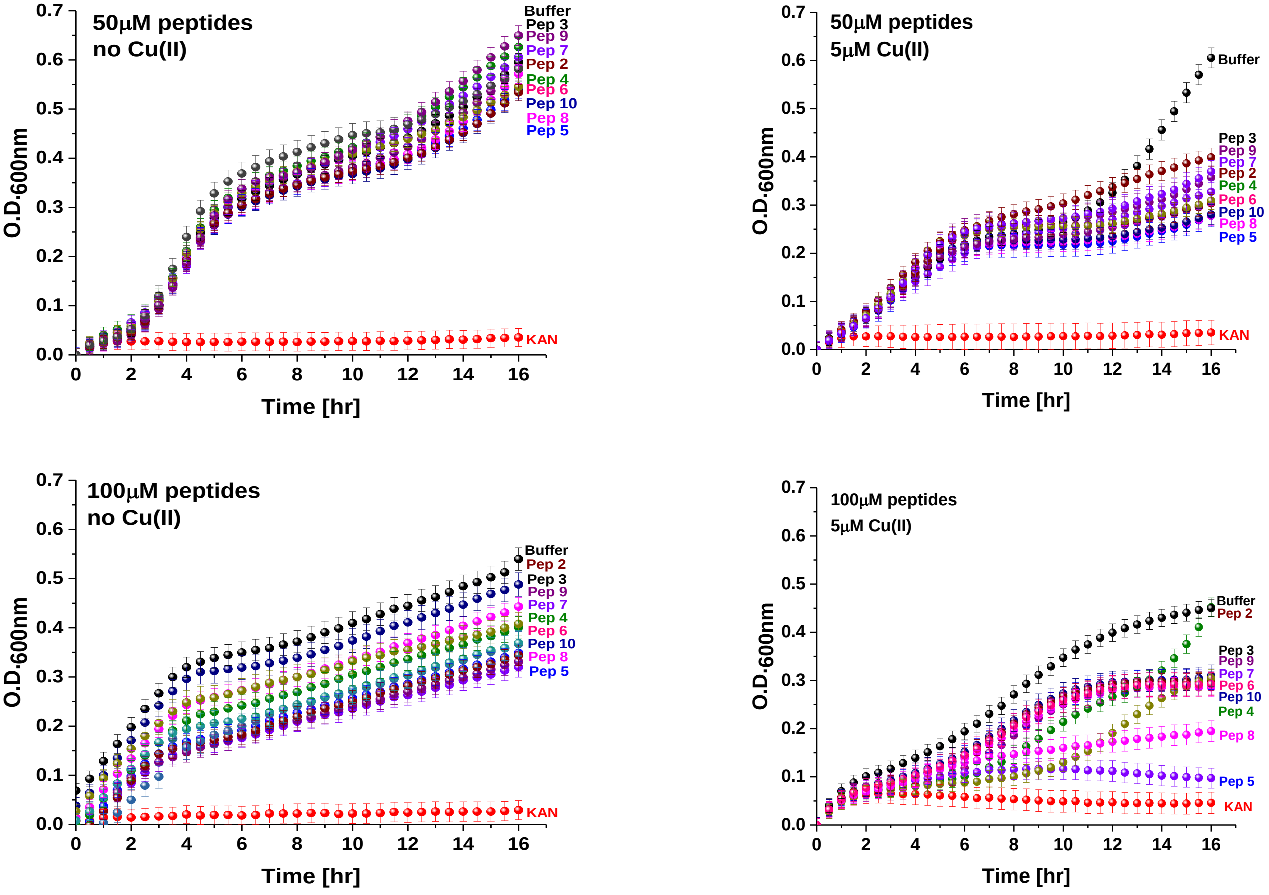

Fig E: E. coli growth experiment. O.D. at 600 nm were measure during 16 hr of incubation with indicated peptides, at concentrations of 50 μM, and 100 μM. All measurements were carried out or in the absence (left panel) or in the presence (right panel) of 5 μM Cu(II).

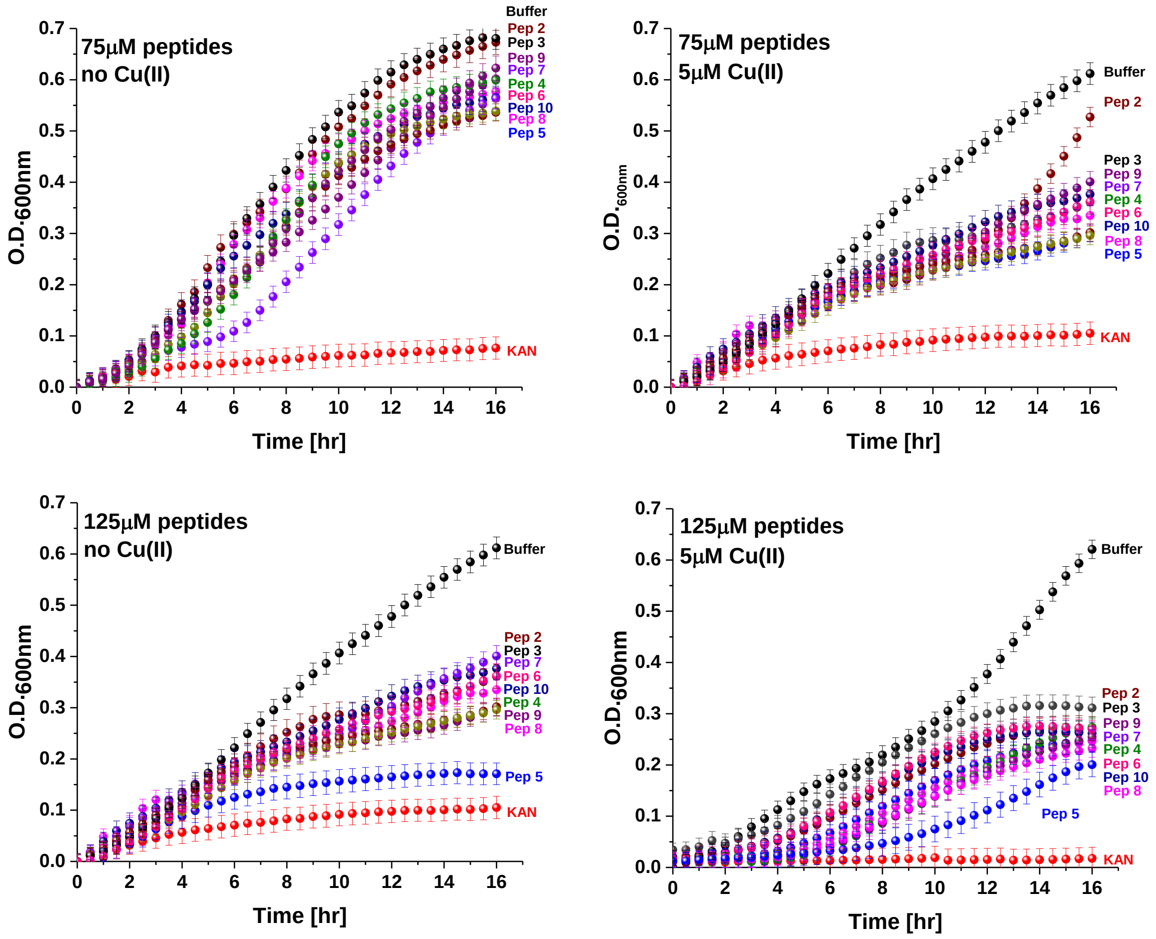

Fig F: E. coli growth experiment. O.D. at 600 nm were measure during 16 hr of incubation with indicated peptides, at concentrations of 75 µM, and 125 µM. All measurements were carried out or in the absence (left panel) or in the presence (right panel) of 5 µM Cu(II).

E. coli viability assay

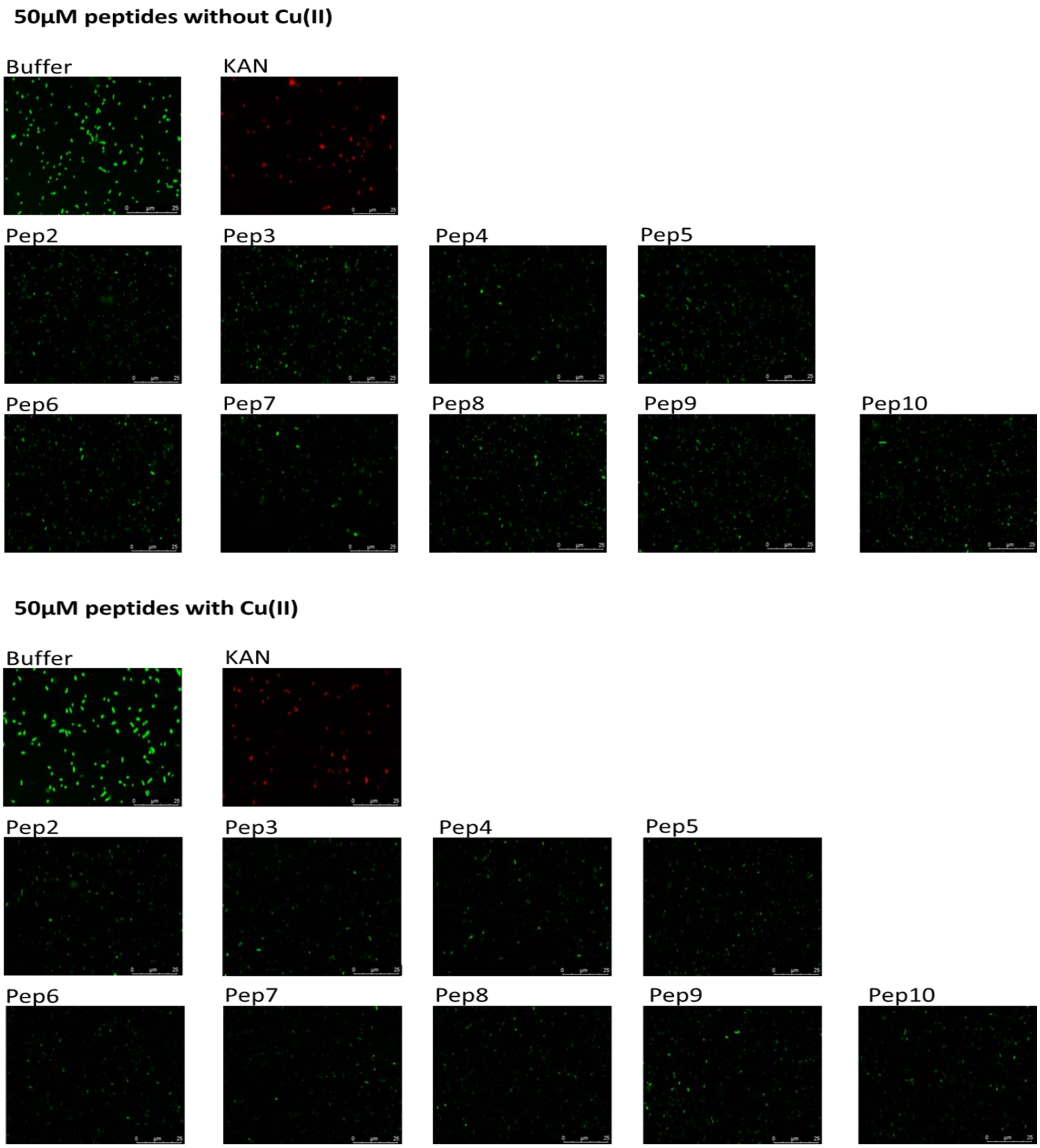

Fig G: Effect of test peptides on the bacteria viability (imaging). Peptide concentration was 50 µM.

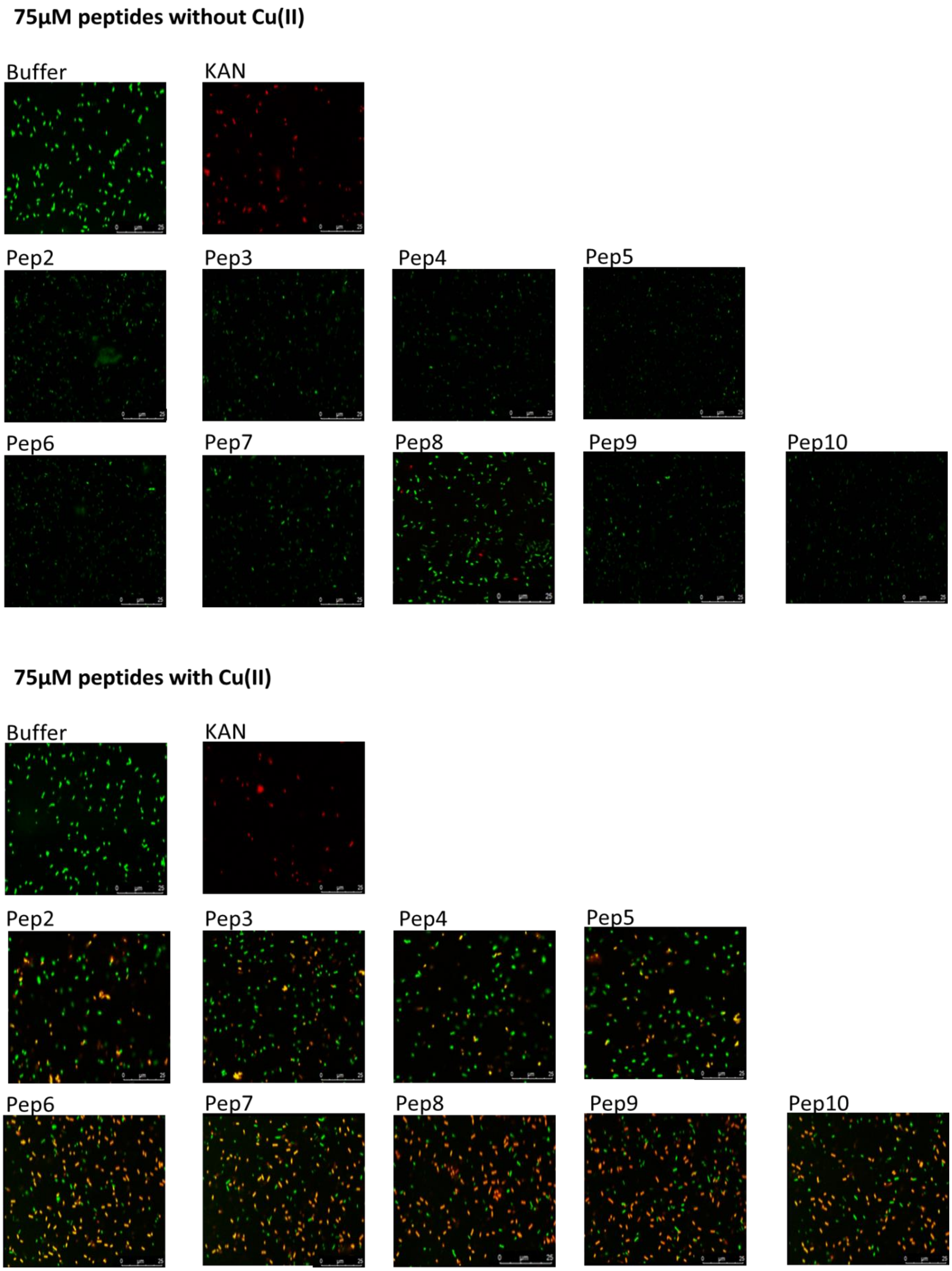

Fig H: Effect of test peptides on the bacteria viability (imaging). Peptide concentration was 75 µM.

100µM peptides without Cu(II)

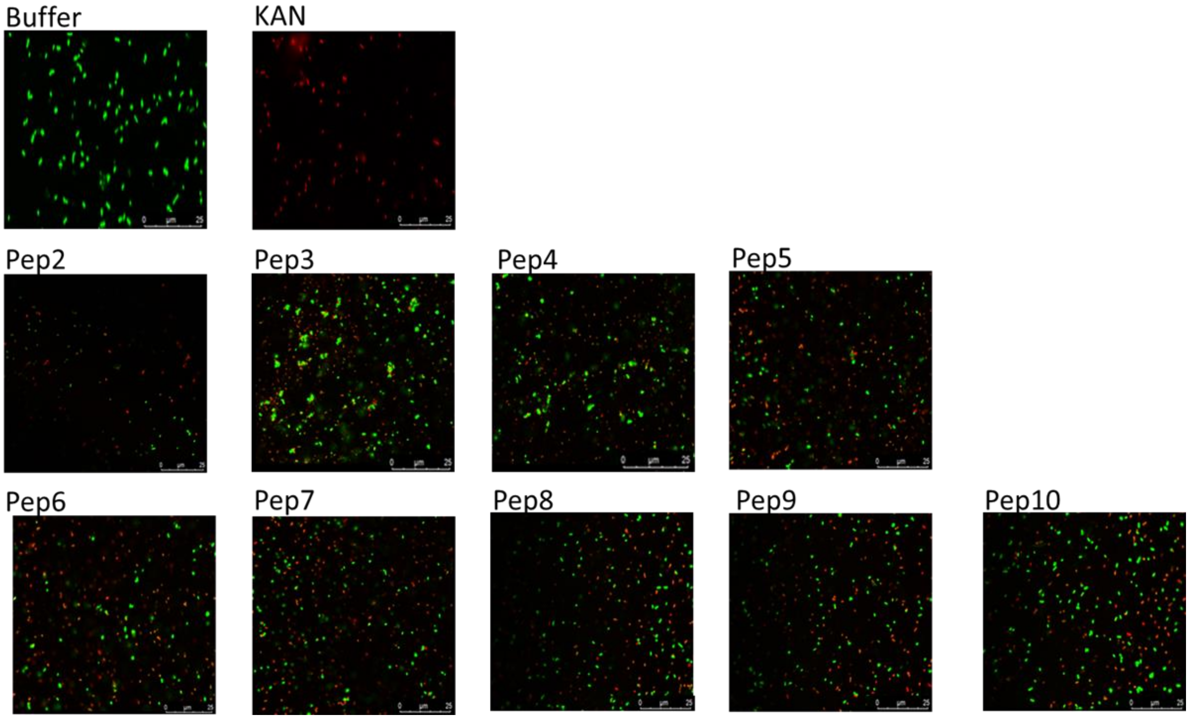

100µM peptides with Cu(II)

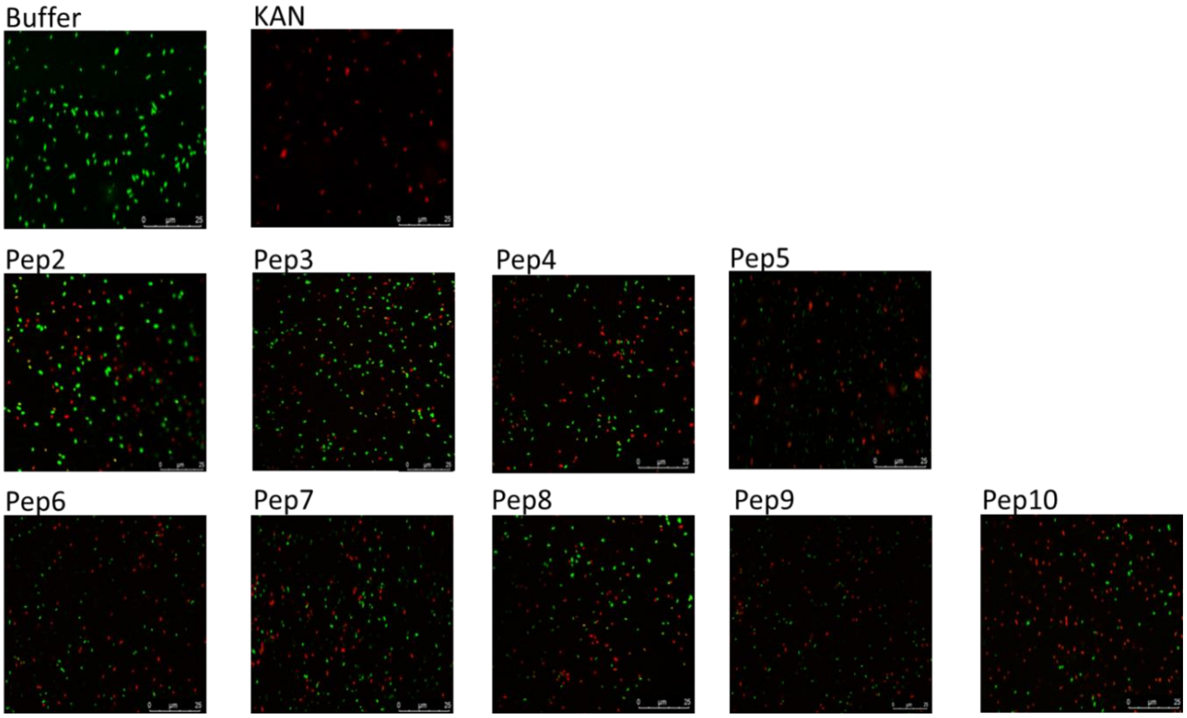

Fig I: Effect of test peptides on the bacteria viability (imaging). Peptide concentration was 100µM.

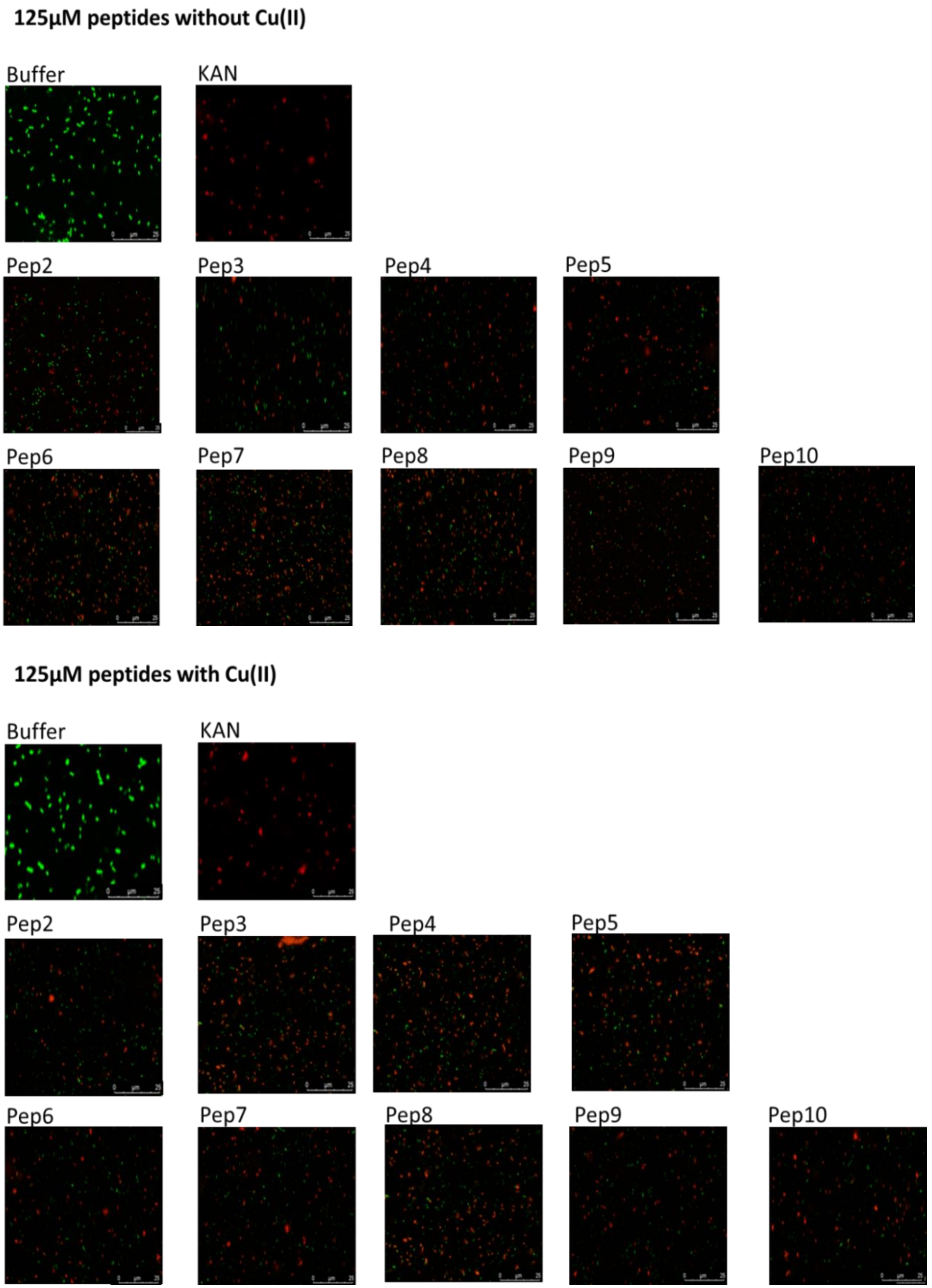

Fig J: Effect of test peptides on the bacteria viability (imaging). Peptide concentration was 125 µM.

In order to better visualize the effect of pep5, Figure S11 presents the transition from mostly live cells (green) to dead cells (red) in the increasing concentration of pep5.

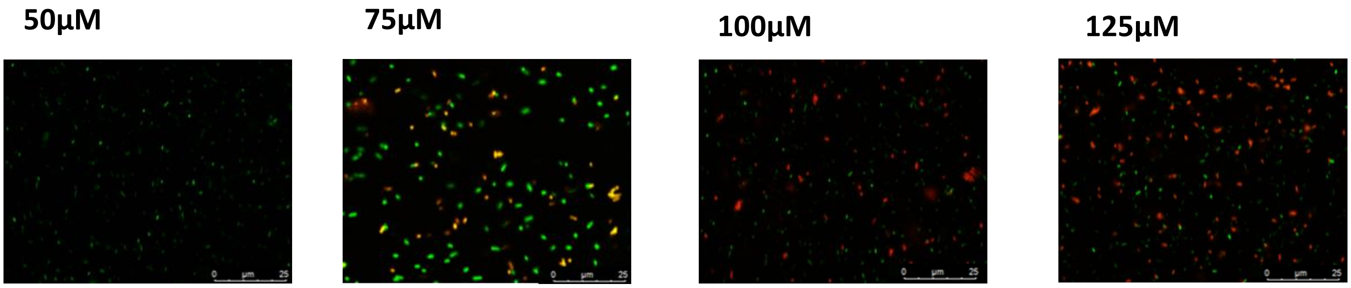

Fig K: Cell viability as a function of pep5 concentration. Cell viability in the presence of pep5 and 5 µM Cu(II) ranging between 50 µM to 125 µM of pep5.

Kirby-Bauer test

10 ml *E. coli* (BL21) were grown up to O.D. 600 nm =0.8, then centrifuged for 15 min 4000 rpm The pellet was suspended in 200 µl LB and cultured on a LB petri dishes. 2 mm radius discs were dipped into specified concentration of the peptide solution (peptide in 0.85% NaCl; sterile) and then placed in the Petri dishes. Petri dishes were left at 37 °C overnight. Figure S12A visualizes the differences in radius formed when different peptide-solution discs were placed in the Petri dish. Table S1 presents the resulting radiuses of bacteria killing. For control experiment, discs were dipped into various concentrations of kanamycin, the bacteria killing radius formed over the disks were measured and presented in Figure S5B.

Table A: Radius formed over petri dishes by peptide-solution discs

	r [mm] 50µM	r [mm] 75µM	r [mm] 100µM	r [mm] 125µM
Buffer	0	0	0	0
Kanamycin	2.5	2.5	2.5	2.5
pep2	0	0	0	0
pep3	0	0	0	0
pep4	0	0	0	0
pep5	0	0.5	1	2
pep6	0	0	0	0
pep7	0	0	0	0
pep8	0	0.5	0.5	1
pep9	0	0.5	0.5	1.5
pep10	0	0	0.5	1.5

A B

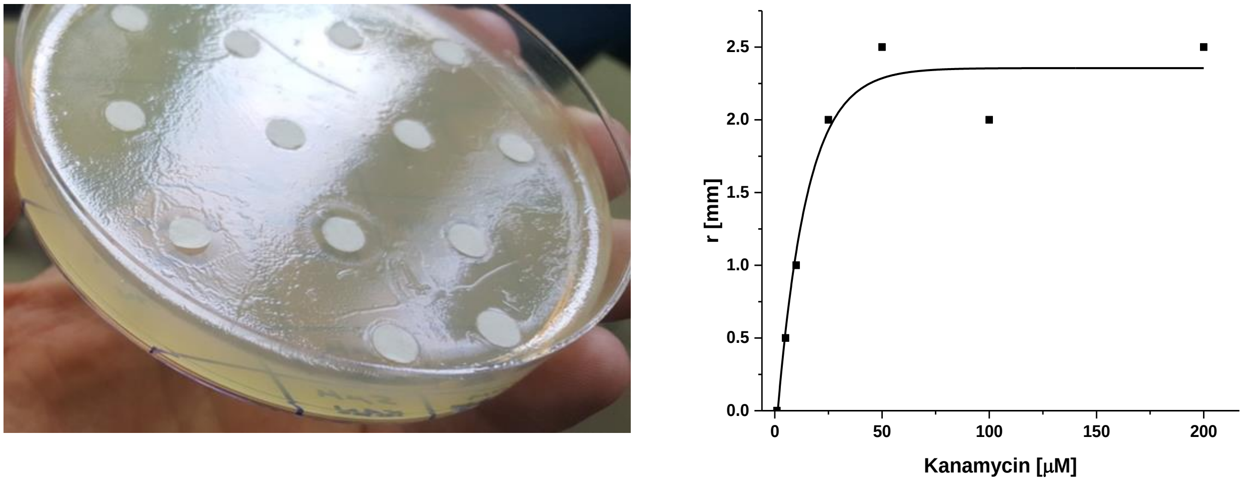

Fig L: Disk diffusion test. A. Visualization of the differences in bacteria toxicity radius after placing peptide-solution-dipped discs B. The bacteria toxicity radius formed by various concentrations of kanamycin.

Cell toxicity (MTT assay)

Cell toxicity (MTT assay) experiments were conducted to verify the effect of pep5 on rat L6 myotubes.

Myotubes were cultured as described in Methods. Pep5 was introduced in various concentrations (three triplicates per concentration) ranging between 0-300 µM and incubated for 24 hr at 37 °C. In addition, compound 8 as a positive control in concertation 100 µM was added. After then medium was taken out and 0.1% of 3-(4,5-dimethylthiazol-2-yl)-2,5-diphenyltetrazolium bromide in PBS buffer was introduced. The myotubes were incubated for 2 hr at 37 °C. The PBS buffer was collected from plates and 200 µl of DMSO were added and incubated with shaking for 30 min at room temperature. Lysates were transferred to 96 well plates and the absorption values were collected using Synergy plate reader at wavelength of 570 nm. Calculations were based on the average of three repetitions. The absorption of the control sample without pep5 was determined as a 100%, all pep5 concentrations were compared against the control sample.

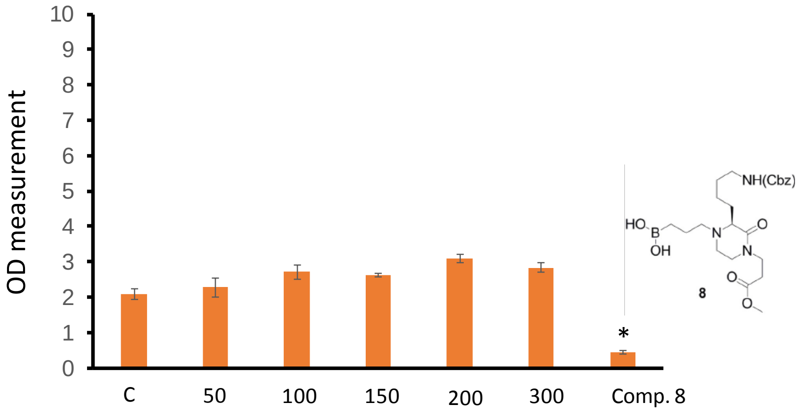

Fig M: Cell toxicity experiments (MTT assay). Absent of the inhibition of L6 myotubes viability in the presence of pep5 gradient concentration. *p≤0.05, MEAN±SE, n=3. Student t-test.

CW-EPR (Continuous-Wave EPR) measurements

CW-EPR spectra were recorded using an E500 Elexsys Bruker spectrometer operating at 9.0–9.5 GHz. The spectra were recorded at room temperature using a microwave power of 20.0 mW, a modulation amplitude of 1.0 G, a time constant of 60 ms, and a receiver gain of 60.0 dB. The samples were measured in 0.8 mm capillary quartz tubes (VitroCom). CusB was labeled at A236C and A248C (identical labeling as the DEER measurements) and measured both in the apo-state (Cu(I)-free) and in the holo-state (Cu(I)-bound) as well as the holo-state in the presence of pep5, pep8, and pep2. CusB presents significant changes in the hyperfine interaction (a_N) upon Cu(I) binding which may be related to the conformational changes the protein undergoes in the environment surrounding the spin label. With the addition of pep5 the hyperfine interaction is similar to the apo-state of CusB indicating on the inhibition of those conformational changes. Pep8 also presented some changes in the hyperfine interaction, yet not as significant as pep5 did. For pep2 no major hyperfine interaction changes were observed compared with the holo-state of CusB.

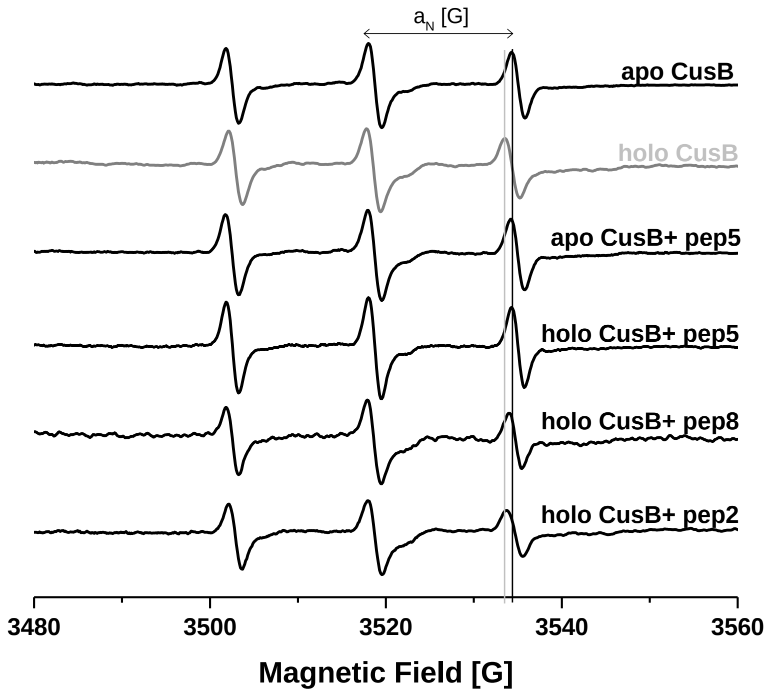

Fig N: CW-EPR measurements. CW-EPR spectra of CusB (labeled at A236C and A248C) for the apo-state, holo-state (grey), apo-state and pep5, holo-state and pep5, holo-state and pep8, and holo-state and pep2.
