# Supplementary material for: Inhibiting the copper efflux system in microbes as a novel approach for developing antibiotics
Source: PLoS One. 2019 Dec 30;14(12):e0227070. doi: 10.1371/journal.pone.0227070 (PMC6936879; doi:10.1371/journal.pone.0227070)
Supplement: Attachment — Submitted filename: answer to reviewers.docx [file pone.0227070.s002.docx]

1. Line 212: BL21 cells not Bl21

Author replay: Corrected

2. Line 215: I do not understand the phrase “..with a script fully described next.” With respect to use of ImageJ software

Author replay: thanks, we deleted this sentence

3. Line 240: Please clarify what is meant by the 13-amino acid peptide containing too many features, I am guessing the authors are referring to features used in the Pharmacore program, but cannot be sure

Author replay: Thank you for the comment, we referred to too many pharmacophore features and added this information to the text in page 9 for clarity.

4. Line 245: I do not see the red circle in Figure 1 indicating the region where the peptide overlaps with the binding site, please include or clarify in figure or caption.

Author replay: Thank you for the comment, we added the missing red circle and also changed the orientation and color coding as suggested in comment No.5.

5. I would suggest using the same orientation and color coding for the protein model in Figures 1 and 2 so the reader can more easily follow the orientation and geometry of the bound peptide

Author replay: Thank you for the comment, we addressed this in the above comment.

6. Lines 261-262: Authors refer to “All ten chose peptides…” being short sequences of the parent peptide (pep1) – in Table 1 there are a total of ten peptides, including the parent peptide. The sentence in lines 261-262 should then read “Nine chosen peptides were short subsequences of the parent peptide (pep1).”

Author replay: we corrected it according to the reviewer’s suggestion

7. Lines 262-263: Should start a new paragraph.

Author replay: done

8. Line 263: “Several peptides were obtained in purity level, which is below 95%” should be re-written

Author replay: we rewrote and clarified this paragraph.

9. Lines 264-265: Poorly written sentence at end of the paragraph.

Author replay: corrected

10. Line 277: Define SOD, superoxide dismutase?

Author replay: we added the definition
11. Line 279-280: Sentence about peptides remaining after incubation is poorly written

Author replay: we corrected it

12. Line 376: C-terminus, singular not plural

Author replay: thanks - corrected

13. Lines 419-420: Statement about peptide not affecting mammalian cells – reference to SI missing?

 Author replay: yes, sorry for that, we added the reference to the S1 file.
